# Accuracy of novel urinary biomarker tests in the diagnosis of prostate cancer: A systematic review and network meta-analysis

**DOI:** 10.3389/fonc.2022.1048876

**Published:** 2022-11-15

**Authors:** Leibo Wang, Wei He, Guanyu Shi, Guoqiang Zhao, Zhuangding Cen, Feng Xu, Wu Tian, Xin Zhao, Chishou Mo

**Affiliations:** ^1^ Surgery, Guizhou Orthopaedic Hospital, Guiyang, Guizhou, China; ^2^ Department of Urology, Fenggang County People’s Hospital, Zunyi, Guizhou, China

**Keywords:** accuracy, prostate cancer, urinary biomarkers, diagnosis, SelectMDx, MIPS

## Abstract

**Objective:**

The purpose of this study was to conduct a network meta-analysis comparing the diagnostic value of different urinary markers for prostate cancer.

**Methods:**

As of June 2022, the literature was retrieved by searching Pubmed, EMBASE, Web of Science databases and other databases. The methodological quality of included studies was assessed using the Cochrane Collaboration’s risk of bias tool, and publication bias was assessed using funnel plots. The surface under the cumulative ranking curve (SUCRA) values ​​was used to determine the most effective diagnostic method and the data were analyzed accordingly using data analysis software.

**Results:**

A total of 16 articles was included including 9952 patients. The ranking results of network meta-analysis showed that the diagnostic performance of the four urine markers Selectmdx, MIPS, PCA3 and EPI was better than that of PSA. Among them, the specificity, positive predictive value and diagnostic accuracy of Selectmdx ranked first in the SUCRA ranking (SUCRA values: 85.2%, 88.3%, 97.1%), and the sensitivity ranked second in the SUCRA ranking (SUCRA value: 54.4%), and the negative predictive value ranked fourth in SUCRA (SUCRA value: 51.6%). The most sensitive screening tool was MIPS (SUCRA value: 67.1%), and it was also the second screening tool ranked higher in specificity, positive predictive value, negative predictive value and diagnostic accuracy (SUCRA value: 56.5%, respectively)., 57.1%, 67.9%, 74.3%). The high negative predictive value SUCRA ranking is EPI (SUCRA value: 68.0%), its sensitivity ranks third (SUCRA value: 45.6%), and its specificity, positive predictive value and diagnostic accuracy are ranked fourth (SUCRA values are: 45%, 38.2%, 35.8%).

**Conclusion:**

According to the network ranking diagram, we finally concluded that Selectmdx and MIPS can be used as the most suitable urine markers for prostate cancer screening and diagnosis. To further explore the diagnostic value of different urinary markers in the screening of PCa patients.

**Systematic Review Registration:**

https://inplasy.com/, identifier INPLASY202290094.

## 1 Introduction

Prostate cancer (PCa) is the most common malignancy in men and the second leading cause of cancer-related death in men worldwide, after lung cancer, with more than 350,000 deaths worldwide in 2018 (1–3). Age and family history are high-risk factors for prostate cancer (4), the risk of prostate cancer rises rapidly after age 50, and approximately one in seven men will be diagnosed with prostate cancer in their lifetime (5, 6). According to the report of the American Cancer Research Center (7), there are about 1.3 million new cases each year, and it is the most common cancer in men in more than half of the countries in the world (105 out of 185 countries), thus becoming a major global public cancer. Hygiene problems seriously affect men’s quality of life and physical and mental health, and place a heavy burden on their families.

Large-scale population screening and early detection are the main preventive measures for PCa (8). Currently, serum prostate-specific antigen (PSA) testing is the most widely used initial screening method for prostate cancer (9). It has been reported that in 22 071 men aged 40 to 84 years with a 10-year follow-up, when the cutoff value was 4.0 ng/ml, the sensitivity of SPSA for detecting prostate cancer was only 46% (10). However, the PSA also lacks sufficient specificity as a screening tool, as elevated serum PSA is not unique to prostate cancer, and a large prostate, digital rectal examination, benign prostatic hyperplasia (BPH), or prostatitis can all lead to false positives, which can lead to false positives. lead to unnecessary overdiagnosis and overtreatment (11). Kishor Mistry et al. conducted a meta-analysis of prostate-specific antigen (PSA) as a PCa screening tool (12, 13), the results showed that the positive predictive value (PPV) of serum PSA was only about 25%, and as many as 75% of patients received unnecessary prostate biopsy. Therefore, prostate cancer screening based on prostate-specific antigen (PSA) detection is controversial, and there is an urgent need to develop new and reliable non-invasive biomarkers for the early diagnosis of PCa.

So is there a more accurate, non-invasive screening and diagnostic option for PCa? Over the past decade, various molecular biomarker tests have been developed as diagnostic tools for the early and non-invasive detection of PCa. Since urine is easy to collect and prostate cells are released directly into the urethra through the prostate catheter after digital rectal examination (DRE) (14). In this context, the detection of urine-related biomarkers obtained in a non-invasive manner has become a better alternative screening tool and has become a research hotspot. Some novel urine markers, such as Progensa Prostate Cancer Antigen 3 (PCA3), SelectMDX, ExoDx Prostate Intelliscore (EPI), Mi-ProstateScore (MIPS), etc., have been gradually used for monitoring and detection of prostate cancer (15, 16). However, data on the diagnostic accuracy by which urine marker regimens are most suitable as screening and diagnostic tools for PCa patients are incomplete, and evidence-based recommendations are still lacking. Therefore, it is necessary to select a reliable and surrogate non-invasive marker to reduce overdiagnosis of suspected PCa patients.

A network meta-analysis(NMA) is an evidence-based technique that uses direct or indirect comparisons to compare the impact of multiple interventions on a disease and to estimate the rank of each measure (17). Therefore, this study pooled existing evidence and used a network meta-analysis to compare different urine markers (Progensa Prostate Cancer Antigen 3 (PCA3), SelectMDX, ExoDx Prostate Intelliscore (EPI), Mi-ProstateScore (MIPS)) to Evaluating and comparing the diagnostic performance of these urine markers in patients with PCa will provide patients and clinicians with more evidence-based data for the disease to guide selection of appropriate diagnostic methods for screening and diagnostic evaluation of patients with prostate cancer.

## 2 Materials and methods

This study was carried out in strict accordance with the preferred reporting items of systematic review and meta-analysis. The protocol has been registered on the INPLASY website (Registration number: INPLASY202290094).

### 2.1 Search strategy

A comprehensive and systematic search was conducted for articles published in Pubmed, embase, Web of Science and CKNI databases from the time the database was established to June 2022. The searched keywords mainly include “Prostatic Neoplasms”, “Urinary biomarkers”, “Progensa PCA3”, “ExoDx Prostate Intelliscore”, “SelectMDx” and “Mi-ProstateScore”. References, and supplementary inclusion of eligible literature. The detailed search strategy (taking Pubmed as an example) is shown in **Supplementary Table 1**.

### 2.2 Inclusion criteria

Participants: Men with elevated PSA levels or abnormal digital rectal examination (DRE) scheduled for prostate biopsy, to provide post-urine or direct urine samples after DRE but before biopsy. 2. Urine markers were used to assist in the diagnosis of prostate cancer; 3. The screening tools included urine markers and no less than two diagnostic methods; 4. The following outcome indicators were reported: true positive (TP), true negative (TN), false positive (FP), false negative (FN), sensitivity (Se), specificity (Sp), accuracy, positive predictive value (PPV) or negative predictive value (NPV). Calculated from known variables (Se and Sp) in cases where NPV, PPV, TP, TN, FP or FN are not reported.

### 2.3 Exclusion criteria

(1) History of prostate cancer, known taking medications that affect PSA levels, previous prostatectomy, or inability to provide post-DRE urine samples; (2) Exclude studies that are not urine markers or studies with less than two diagnostic methods; (3) Lack of clear inclusion and exclusion criteria in the study; (4) Letters to the editor, editorials, research protocols, case reports, brief communications, non-clinical studies, missing research data, and duplicate published articles, etc.

### 2.4 Data extraction

Lb W imported the search results into the document manager EndNoteX9. After eliminating duplicate documents, Lb W and WH screened the documents by browsing titles, abstracts, and full-text reading, respectively. Lb W and Gq Z independently extracted data from the literature that met the inclusion and exclusion criteria. The main extracted information included: study authors, publication time, country, number of patients, age, tumor stage and grade, cutoff values of biomarkers, and primary outcome measures Se, Sp, TP, FP, FN, and TN. If there is any disagreement, it will be discussed with Gy S according to the screening criteria.

### 2.5 Literature quality assessment

Zd C and FX used the Diagnostic Accuracy Research Quality Assessment Tool (QUADAS-2) (18) to conduct independent quality evaluation, and checked the evaluation results, and any disagreements were resolved by WT. The scale evaluation includes the risk of bias and clinical applicability evaluation. The risk of bias consists of four parts: case selection, trials to be evaluated, gold standard, case flow and progress. All components were assessed for risk of bias, and the first 3 components were also assessed for clinical applicability. The risk of bias was rated as “low”, “high” or “uncertain”.

### 2.6 Data analysis

We use state software (version 15.1) to aggregate and analyze NMAs using a Markov Monte Carlo simulation chain in a Bayes-based framework (19).

The Stata software will present and describe a network diagram of the different urine markers. In the resulting network graph, each node represents a different urine marker, and the lines connecting the nodes represent direct head-to-head comparisons between urine markers. The size of each node and the width of connecting lines are proportional to the number of studies (20)。To help explain the diagnostic performance, the Surface Under the Cumulative Ranking Curve (SUCRA) (21) was used to calculate the probability of each urine marker and was the most efficient diagnostic method based on a Bayesian approach using probability values, the greater the SUCRA value, the higher the diagnostic performance rating of the urine marker. This study used a funnel plot test to determine publication bias (22). If the inverted funnel plot is asymmetric, publication bias may exist; otherwise, there is no apparent publication bias.

## 3 Results

### 3.1 Study and identification and selection

**Figure 1:**
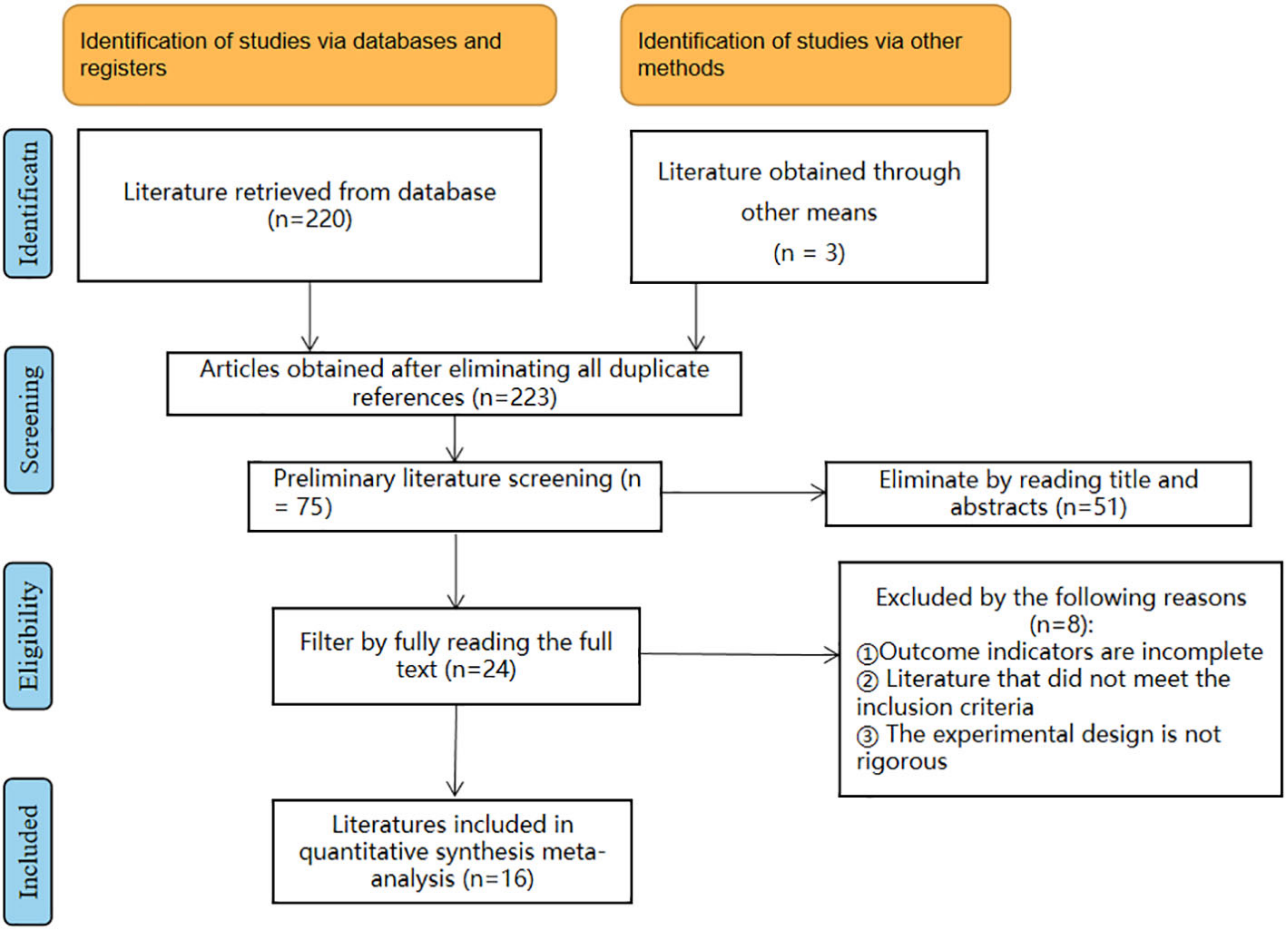
Flow diagram of literature selection.

According to the literature search of the database according to the search strategy, a total of 223 literatures was retrieved from the database. After removing duplicates, 75 articles remained. Then, by reading the titles and abstracts, 51 articles were excluded, and 8 of the remaining 24 articles were deleted due to incomplete outcome indicators, literature that did not meet the inclusion criteria, and imprecise experimental design. Finally, 16 articles were included in the meta-analysis, and the detailed literature screening process is shown in **Figure 1**.

The characteristics and demographic data of the included studies are shown in **Table 1**. Sixteen studies includingwas retrieved were included in the quantitative analysis. All studies in the quantitative analysis reported the diagnostic value of urine markers in prostate cancer: 10 studies on PCA3, 3 studies on Selectmdx, 2 studies on MIPS, and 1 study on ExoDx Prostate Intelliscore.

**Table 1:** Characteristics of the studies included in the meta-analysis.

Author	Country	Year	Number of patients	Study design	Age (mean+SD)	Tumor stage	1eason score	Sensitivity	Specificity	NPV	PPV	Cutoff of biomarker
Goode RR (23)	USA	2012	289	retrospective	IQR:(66)41–90	T1c 60 (67.4) T2a 15 (16.9) T2b 5 (5.6) T2c 9 (10.1)	3+3 59 (62.1%) 3+4 19 (20.0%) 4+3 6 (6.3%) 4+4 10 (10.5%) 4+5 1 (1.1%)	PCA3:0.67 PSA:0.61	PCA3:0.79 PSA:0.47	PCA3:0.71 PSA:0.55	PCA3:0.76 PSA:0.54	PSA:4.0 PCA3: 35
Tinzl M (24)	Austria	2004	201	prospective	mean:66	<T2:53 (72%); T3:21 (28%)	<7 45 (61%) 7 14(19%) >7 15 (20%)	PCA3:0.82 PSA:0.87	PCA3:0.76 PSA:0.16	PCA3:0.81 PSA:0.55	PCA3:0.77 PSA:0.51	PSA:4.0 PCA3:NA
Roobol MJ (25)	Netherlands	2010	721	prospective	IQR:70.23(63.7–74.0)	NR	NR	PCA3:0.67 PSA:0.61	PCA3:0.56 PSA:0.16	PCA3:0.64 PSA:0.51	PCA3:0.61 PSA:0.55	PSA:≥3.0 PCA3:≥10
Taille Adl (26)	France	2011	516	prospective	63.0 ± 7.6	T1c 140 (86) T2 20 (12) T3a 2 (1.2)	<6 1 (0.5) 6 108 (52) 7 87 (42) >7 11(5.3)	PCA3:0.64 PSA:0.91	PCA3:0.76 PSA:0.16	PCA3:0.68 PSA:0.64	PCA3:0.73 PSA:0.52	PSA: 4.0 PCA3: 35
Vlaeminck-Guillem V (27)	France	2011	240	prospective	63 ± 7	NR	NR	PCA3:0.60 PSA:0.66	PCA3:0.68 PSA:0.32	PCA3:0.61 PSA:0.46	PCA3:0.67 PSA:0.52	PSA: 4.0 PCA3: 35
C F Ng (28)	China	2012	47	prospective	IQR:70 (59-84)	T1c 9 T2 6 T3 2	<7 7 ≥7 10	PCA3:0.71 PSA:1	PCA3:0.92 PSA:0.21	PCA3:0.76 PSA:1	PCA3:0.90 PSA:0.56	PSA:>4.0 PCA3: NA
Ouyang B (29)	USA	2009	92	prospective	69.5 ± 10.5	NR	5-6 35 (83.7%) 7-8 7 (16.3%)	PCA3:0.72 PSA:0.77	PCA3:0.59 PSA:0.45	PCA3:0.71 PSA:0.69	PCA3:0.61 PSA:0.55	PSA: 4.0 PCA3: 19.9
Ramos CG (30)	Chile	2012	64	prospective	62.1± 8.4	NR	6 13 (52%) 7 5 (20%) 8 5 (20%) 9 2 (8%)	PCA3:0.52 PSA:0.83	PCA3:0.87 PSA:0.21	PCA3:0.64 PSA:0.55	PCA3:0.80 PSA:0.51	PSA:>4 PCA3:>35
van Gils MP (31)	Netherlands	2007	534	prospective	64.3 ± 7.2	NR	NR	PCA3:0.65 PSA:0.65	PCA3:0.66 PSA:0.47	PCA3:0.65 PSA:0.57	PCA3:0.66 PSA:0.55	PSA: NA PCA3: 58
Fradet Y (32)	Canada	2004	443	prospective	IQR:64(40-87)	NR	6-7 319(72%)	PCA3:0.66 PSA:0.58	PCA3:0.89 PSA:0.91	PCA3:0.72 PSA:0.68	PCA3:0.86 PSA:0.87	PSA:4 PCA3: NA
McKiernan J (33)	USA	2018	503	prospective	IQR:64 (59–69)	NR	3+3 111 (22.1) 3+4 86 (17.1) 4 + 3 26 (5.2) 8 26 (5.2) >8 20 (4)	EPI:0.9 PSA:0.9	EPI:0.39 PSA:0.2	EPI:0.79 PSA:0.66	EPI:0.59 PSA:0.53	PSA : NA EPI:15.5
Sanda MG (34)	USA	2017	561	prospective	IQR:60 (33-79)	NR	≤6 98 (18.99) ≥7 156 (30.23)	MIPS:0.91 PCA3:0.97 PSA:0.91	MIPS:0.35 PCA3:0.18 PSA:0.17	MIPS:0.79 PCA3:0.84 PSA:0.65	MIPS0.58 PCA3:0.54 PSA:0.52	MIPS:>8 PCA3:>20 PSA:>10
Salami SS (35)	USA	2013	45	prospective	IQR: PCa:64(56–70) NPCa:65(58–71)	NR	≥7 7(47%)	MIPS:0.9 PCA3:0.93 PSA:0.4	MIPS:0.8 PCA3:0.37 PSA:0.93	MIPS:0.94 PCA3:0.91 PSA:0.76	MIPS:0.69 PCA3:0.42 PSA:0.75	MIPS:NA PCA3:NA PSA:10
Van Neste L (36)	Netherlands	2016	519	prospective	IQR:65(60–70)	NR	6 103 (48.6) 7 58 (27.4) 8-10 51 (24.1)	selectmdx:0.91 PCA3:0.91	selectmdx:0.36 PCA3:0.2	selectmdx: 0.8 PCA3:0.69	selectmdx:0.59 PCA3:0.53	Selectmdx:27.5 PCA3: 35
Busetto GM (37)	Italy	2021	52	prospective	64 ± 8.7	NR	NR	selectmdx:0.94 PCA3:1	selectmdx:0.91 PCA3:0.17	selectmdx: 0.97 PCA3:1	selectmdx:0.84 PCA3:0.37	Selectmdx:NA PCA3: NA
Fiorella D (38)	Spain	2021	86	prospective	65.30 ± 6.90	NR	3+3 8 2 (95.35%) ≥3+4 4 (4.65%)	selectmdx:0.7 PCA3:0.51	selectmdx:0.67 PCA3:0.74	selectmdx:0.6 PCA3:0.60	selectmdx:0.68 PCA3:0.67	Selectmdx:5 PCA3: 65

### 3.2.Quality assessment of the included studies

**Figure 2:**
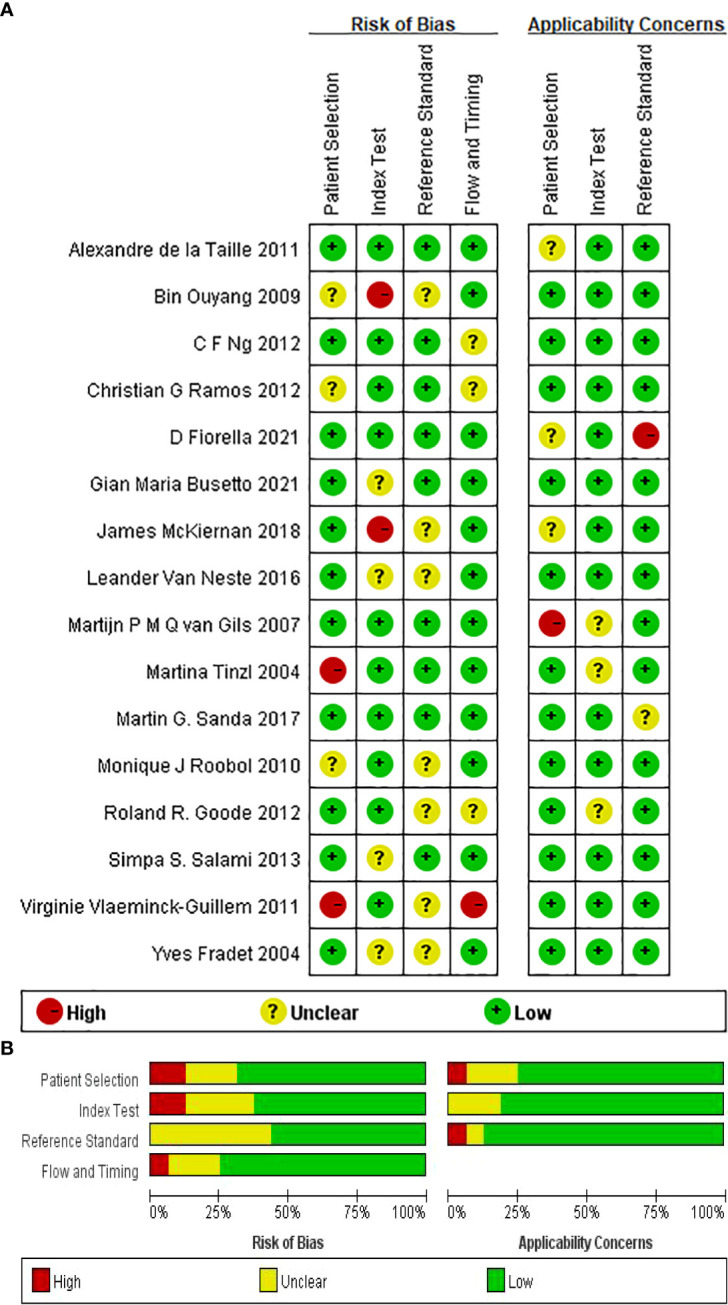
**(A)** Summary of risk of bias for each studies. **(B)** Proportion of risk of bias for all domains.

**Figure 3:**
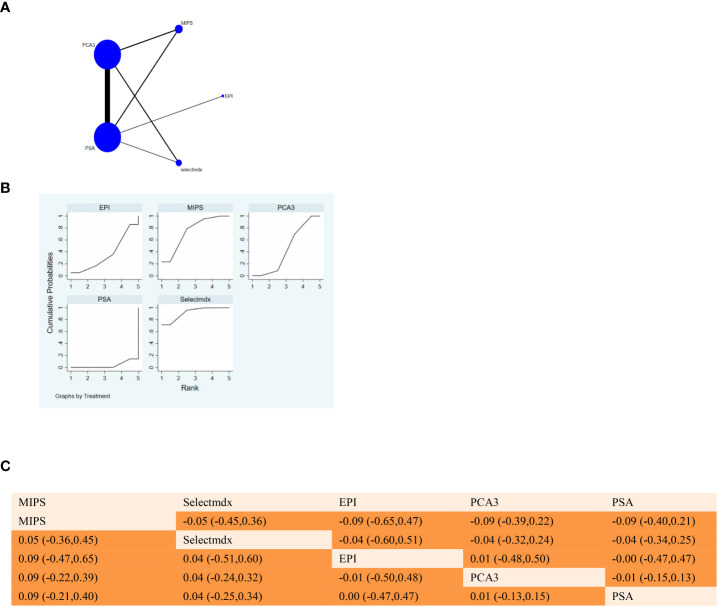
Proportion of risk of bias for all domains. **(A)** NMA figure for SE. **(B)** SUCRA plot for Sensitivity. **(C)** League table on Sensitivity.

We included 1 retrospective study (23) and 15 prospective studies out of 16 articles (24–32, 34–39). We performed a network meta-analysis using STATA version 15.1 and assessed 16 papers for quality, risk of bias, and applicability issues using QUADAS-2. The overall quality of the article is satisfactory. In terms of personnel selection, 3 of the 16 studies had a unclear risk of bias and 2 had a high risk of bias, and patients themselves or their relatives had to sign an informed consent before urine markers were tested in these studies. In terms of indicator detection, there were 4 studies with unclear risk of bias and 2 studies with a high risk of bias. In terms of reference standard bias, seven studies had a unclear risk of bias. Three articles had an uncertain risk of bias with respect to follow-up time. In terms of applicability, there was a high risk of bias in patient selection and reference standards and there was no increased risk of bias in index testing (**Figures 2**, **3** for details).

### 3.3 Network meta-analysis

**Figure 4:**
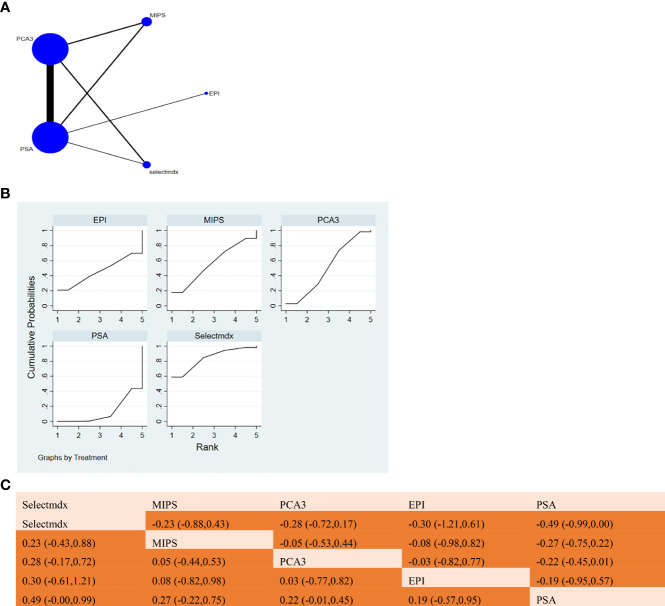
**(A)** NMA figure for Specificity. **(B)** SUCRA plot for Specificity. **(C)** League table on Specificity.

**Figure 5:**
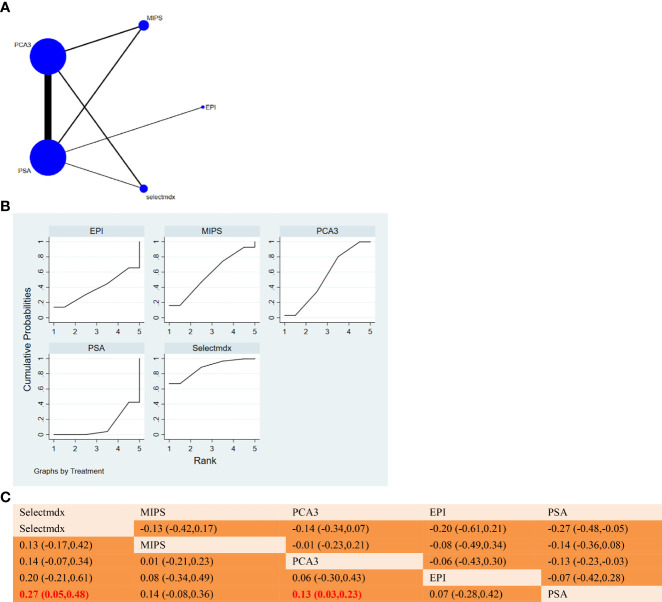
**(A)** NMA figure for PPV. **(B)** SUCRA plot for PPV. **(C)** League table on PPV.

**Figure 6:**
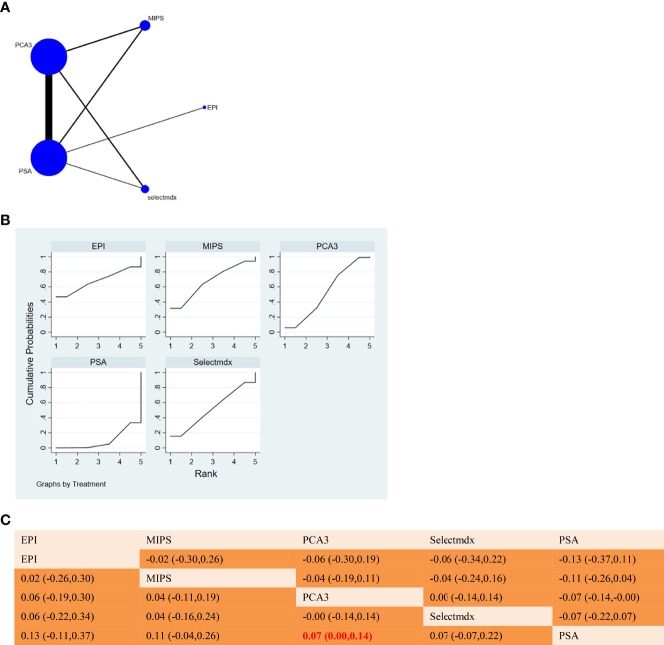
**(A)** NMA figure for NPV. **(B)** SUCRA plot for NPV. **(C)** League table on NPV.

**Figure 7:**
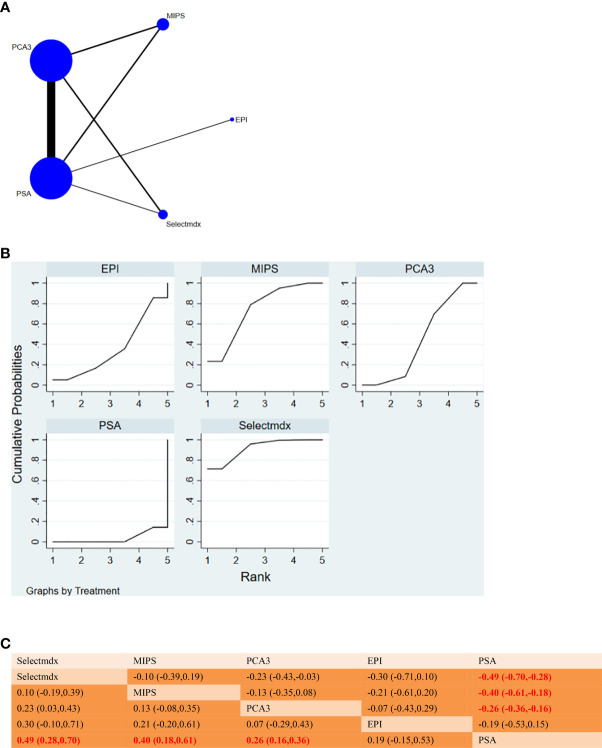
**(A)** NMA figure for Accuracy. **(B)** SUCRA plot for Accuracy. **(C)** League table on Accuracy.

The full NMA figure will be shown in **Figures 3A**, **4A**, **5A**, **6A**, **7A**.

#### 3.3.1 Sensitivity

The P values of indirect and direct comparisons between all the studies were tested for consistency and inconsistency, and the P values were all greater than 0.05, indicating that the effect of inter-study consistency was acceptable.

The results of network meta-analysis showed that compared with serum PSA, MIPS [MD = 0.09, 95% CI: 0.40, 0.21)], Selectmdx [MD = 0.04, 95% CI: 0.34, 0.25)], EPI [MD = 0.00, 95% CI: 0.47, 0.47)], PCA3 [MD = -0.01, 95%CI:(-0.15,0.13)] was not significantly different from PSA in terms of sensitivity. In the cumulative area under the ranking curve (SUCRA), MIPS(67.1%) and Selectmdx(54.4%) ranked the top two in terms of sensitivity probability (as shown in **Figure 3B**). **Figure 3C** shows a comparison between these two different detection measures.

#### 3.3.2 Specificity

The results of network meta-analysis showed that compared with serum PSA, Selectmdx [MD=0.49, 95%CI: (-0.00,0.99)], MIPS [MD=0.27,95%CI: (-0.22,0.75)], EPI [MD=0.19, 95%CI: (-0.57,0.95)], PCA3 [MD=0.22, 95%CI: (-0.01,0.45)] were not significantly different in specificity. In the cumulative ranking area under the curve (SUCRA), the different markers were ranked as Selectmdx(85.2%)>MIPS(56.5%)>PCA3(51.5%)>EPI(45.0%)>PSA(11.8%) (as shown in **Figure 4B**) Show). **Figure 4C** will show a comparison between these two different detection measures.

#### 3.3.3 PPV

The results of network meta-analysis showed that in terms of negative predictive value, there was a significant difference between Selectmdx and serum PSA [MD=0.27, 95%CI: (0.05, 0.48)], and there was also a significant difference between PCA3 and serum PSA [MD = 0.13,95%CI:(0.03,0.23)]; MIPS[MD=0.14,95%CI:(-0.08,0.36)], EPI[MD=0.07,95%CI:(-0.28 compared to PSA, 0.42)] was not significantly different in terms of negative predictive value. The order of PPV for diagnosis of prostate cancer from high to low was: Selectmdx(88.3%)>MIPS(57.1%)>PCA3(54.4%)>EPI(38.2%)>PSA(12.0%). (**Figure 5B**). **Figure 5C** will show a comparison between these two different detection measures.

#### 3.3.4 NPV

All p-values for indirect and direct comparisons between studies examined agreement and inconsistency, and p-values were all greater than 0.05, indicating that the effect of between-study agreement was acceptable.

The results of the network meta-analysis showed that compared with PSA, MIPS [MD= 0.11, 95%CI: (-0.04, 0.26)], EPI [MD= 0.13, 95%CI: (-0.11, 0.37)], Selectmdx[MD=0.13, 95%CI:(-0.11,0.37)] no significant difference in positive predictive value; heterogeneity between PCA3 and serum PSA [MD=0.07,95%CI:(0.00,0.14)]. According to the SUCRA curve, it can be concluded that the PPV of different markers in the diagnosis of prostate cancer is from high to low: EPI(68.0%)>MIPS(67.9%)>PCA3(53.0%)>Selectmdx(51.6%)>PSA(9.6%) (**Figure 6B**). **Figure 6C** will show a comparison between these two different detection measures.

#### 3.3.5 Accuracy

The results of the network meta-analysis showed that compared with PSA, Selectmdx [MD= 0.49, 95%CI: (0.28, 0.70)], MIPS [MD= 0.40, 95%CI: (0.18, 0.61)], PCA3 [MD= 0.26,95%CI:(0.16,0.36)] There was heterogeneity in diagnostic accuracy, EPI was not significantly different from PSA in terms of diagnostic accuracy [MD=0.19,95%CI:(-0.15,0.53)]. According to the SUCRA curve, it can be concluded that the diagnostic accuracy of different urine markers in the diagnosis of prostate cancer is Selectmdx(97.1%)>MIPS(74.3%)>PCA3(44.6%)>EPI(35.8%)>PSA(3.6%) (**Figure 7B**). **Figure 7C** will show a comparison between these two different detection measures.

### 3.4 Publication bias test

**Figure 8:**
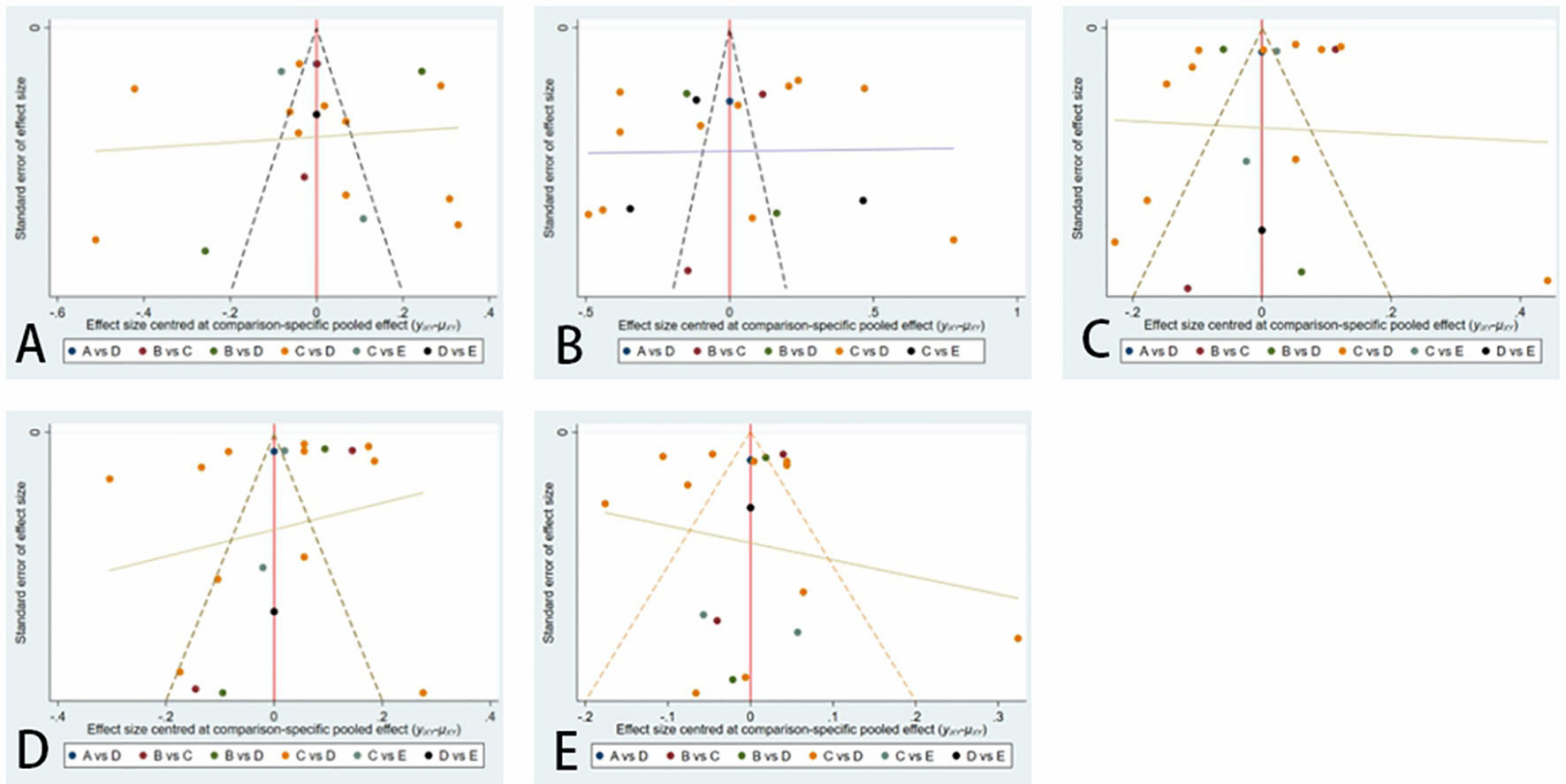
Funnel plot on publication bias.

We constructed separate funnel plots for all outcome measures to test for possible publication bias. Visual inspection of the funnel plot did not reveal any significant publication bias. The details are shown in **Figure 8**.

## 4 Discussion

In this study, we compared the diagnostic value of different novel urine biomarker tests for prostate cancer screening. Sixteen studies were included, with 5 different assays, including serum PSA, and 9952 patients were included in the quantitative analysis. Our research shows that Selectmdx, MIPS, PCA3, and EPI are superior to PSA in terms of sensitivity, specificity, positive predictive value, negative predictive value and diagnostic accuracy. As the most suitable urine marker for PCa screening diagnosis. To our knowledge, several authors have published meta-analyses of two or more urine markers for prostate cancer diagnosis, But our study is the first to provide a comprehensive network meta-analysis of diagnostic tests for these urine markers.

Although serum PSA has been widely used for early screening of prostate cancer for a long time. The results of network meta-analysis showed that the diagnostic performance of Selectmdx, MIPS, PCA3 and EPI was better than PSA. Among them, Selectmdx ranked first in specificity, positive predictive value and diagnostic accuracy in SUCRA ranking, sensitivity ranked second in SUCRA and negative predictive value ranked fourth in SUCRA ranking.Selectmdx (37, 38) is a test that combines urinary biomarkers (DLX1, HOXC6) and clinical risk factors (age, PSA, prostate volume, family history, DRE) and is commonly used to predict abnormally elevated PSA levels of patients had high-grade PCa (Gleason score ≥7) on biopsy. Leyten GH et al (40) showed that urine three gene sets (HOXC6, TDRD1 and DLX1) showed higher accuracy than PSA and Progensa PCA3 tests in detecting prostate cancer (AUC, 0.77 vs. 0.72 vs. 0.68, respectively), DLX1 and HOXC6 may be involved in the pathogenesis and invasiveness of PCa。Van Neste et al (36) collected post-DRE urine samples from 905 patients from two prospective multicenter studies, and the assay was developed using a training cohort of 519 patients and subsequently evaluated in an independent cohort (n=386). The clinically validated AUC of DLX1 and HOXC6 for the prediction of high-grade PCa was 0.76, with a sensitivity of 91%, a specificity of 36%, an NPV of 94%, and a PPV of 27%. In this model, the validation cohort had an AUC of 0.86, a 42% reduction in total biopsies, a 53% reduction in unnecessary biopsies, and an NPV of 98% for high-grade PCa. These findings demonstrate the high diagnostic accuracy and acceptability of Selectmdx in prostate cancer patients. In addition, Maggi M et al. (41) evaluated the diagnostic accuracy of SelectMDx in predicting prostate cancer on prostate biopsy and its association with multiparametric magnetic resonance imaging (mpMRI) in a prospective multicenter study involving 310 patients who underwent mpMRI and SelectMDx before prostate biopsy. The results showed that the sensitivity and specificity of SelectMDx and mpMRI in predicting biopsy PCa were 86.5% vs. 51.9%, 73.8% vs. 88.3%, 87.1% vs. 61.3%, respectively, in predicting biopsy clinically significant prostate cancer (csPCa). 63.7% vs. 83.9%. SelectMDx was found to be a good predictor of PCa, whereas with regard to the csPCa assay, it was shown to be less effective, showing mpMRI-like results, and by analytically evaluating strategies, determining the best diagnosis to avoid unnecessary biopsies Strategy, SelectMDx can be used as a reliable detection method after initial negative mpMRI. The final recommendation is that biopsy should be performed in all cases with an mpMRI Prostate Imaging Reporting and Data System (PI-RADS) score of 4-5, as well as those with a previous PI-RADS score of 1-3 and a positive SelectMDx score. A recent UK cost-effectiveness study (42) demonstrated a savings of €128 (US$143) per patient and a 0.025-year increase in quality of life at a diagnostic sensitivity cutoff of 95.7% for high-grade prostate cancer compared to screening for prostate cancer using PSA alone. Therefore, while considering sensitivity and specificity, SelectMDx has the best diagnostic performance and can be recommended as a promising screening tool in PCa detection.

The ranking results showed that the most sensitive screening tool was MIPS, and it was also the second screening tool ranked higher in specificity, positive likelihood ratio, negative likelihood ratio, and diagnostic accuracy. The highest negative predictive value SUCRA is EPI, its sensitivity ranks third, and its specificity, positive predictive value and diagnostic accuracy ranks fourth, and the subsequent screening method is PCA3.MIPS (34) is a risk assessment method developed by the University of Michigan that combines urine transmembrane serine protease 2 (TMPRSS2):ERG (Ets-related gene), PCA3 and serum PSA to predict biopsy-positive risk assessment to predict any prostate at the time of biopsy cancer and high-grade prostate cancer risk. In 2005, Tomlins and Chinnaiyan (43) reported a unique gene fusion (TMPRSS2:ERG gene fusion), an abnormal fusion of the TMPRSS2 gene (an androgen-regulated transcriptional promoter) and the ERG oncogene, which usually occurs in in prostate cancer. TMPRSS2:ERG is detectable in urine after DRE, is highly specific for PCa, and is found in approximately 50% of PCa cases in Caucasian males (44).Prostate cancer antigen 3 (PCA3) (45, 46), also known as DD3, is a prostate-specific non-coding messenger RNA (mRNA) located on the long arm of chromosome 9, highly overexpressed in PCa, present in 95% of prostate cancers are barely detectable in normal tissue and other prostate-related diseases. Combining PCA3 with TMPRSS2:ERG can significantly improve the screening power of PCa, and Robert et al. (47) have shown that the combination of these two markers is more sensitive for PCa diagnosis than TMPRSS2:ERG alone (sensitivity: 93.6% vs. 45.8%), while also maintaining high specificity in prostate tissue (98.8% vs. 97.5%).Salami et al (35) also demonstrated that the combination of these two markers improved their ability to predict prostate biopsy results (AUC=0.88). Therefore, combined urine testing of T2:ERG and PCA3 can avoid unnecessary prostate biopsies while maintaining robust sensitivity for detecting aggressive prostate cancer, saving healthcare costs.

It is worth mentioning that the PCA3 test (48) was the first urine biomarker test approved by the U.S. Food and Drug Administration (FDA) in 2012 for use in patients over the age of 50 with elevated sPSA levels and a history of negative prostate biopsy. Of men, the cutoff value was 25 to decide whether a repeat prostate biopsy was needed. Several previous studies have demonstrated (32, 49) that PCA3 testing is superior to serum PSA testing in predicting positive prostate biopsy, and Lee et al (50) also included 54 studies in a meta-analysis involving 17 575 patients, The overall sensitivity, specificity, and diagnostic odds ratio of the PCA3 score were 71% (95% CI: 67%-74%), 68% (95% CI: 63%-74%), and 5.28 (95% CI: 4.28-74%), respectively 6.51), the pooled AUC was 0.75 (95% CI: 0.71 to 0.79).The final study showed that PCA3 detection was superior to serum PSA in diagnosing PCa. However, the choice of the cutoff value is still controversial, and many studies (49, 51) suggest that the cutoff value of 35 can be used as a better cutoff point. A study by Marks et al (49) evaluated different PCA3 score cutoffs: 10 and 35, for which the sensitivity was 87% and 58%, and the specificity was 28% and 72%, respectively. Results showed that a PCA3 score of 35 could provide the best balance between sensitivity (58%) and specificity (72%) for diagnosing PCa, and was superior to PSA. In a meta-analysis of the diagnostic accuracy of PCA3 before prostate biopsy (51), when the cutoff value was 35, AUC = 0.734, with a sensitivity of 69% and a specificity of 65%. These results also support that cutoff = 35 has a greater clinical utility than 25 (FDA approved). In addition, a recent study provided evidence (26) that, when the cutoff value was 20, PCA3 testing was helpful in the decision to perform an initial biopsy, and for men who had not undergone a biopsy and had a PSA level of 2.5-10.0 ng/ml, 40% of biopsies will be avoided, and 95% of men with Gleason ≥7 PCa have higher PCA3 scores. What is currently controversial is the optimal cutoff value of PCA3 score. Some studies have pointed out (34, 52) that there are certain limitations when the PCA3 score takes a single cutoff value. When the PCA3 score takes a lower cutoff value, higher negative Predictive value; While taking a higher cutoff value, a higher positive predictive value can be obtained; when the score lies in between, there is a “grey area”, which may be overcome by combining other tumor markers, such as T2: ERG.

Although the negative predictive value of EPI ranks the highest, it has certain limitations due to the relatively low ranking of sensitivity, specificity, positive predictive value, and diagnostic accuracy, and only 1 included literature.EPI (53) obtained the EPI score by detecting the expression levels of PCA3, ERG, and SPDEF in urinary exosomes. As the first FDA-approved exosome liquid biopsy product, this is also an important milestone for exosomes toward clinical application. The EPI score is mainly aimed at PSA screening gray zone 2-10ng/ml prostate cancer risk patients to exclude unnecessary biopsy. In contrast to other urinary tumor markers for PCa, the ExoDx Prostate Test does not require prostate massage prior to urine collection (54). According to the results of a prospective study published by Mckiernan et al (33), setting the threshold of EPI at 15.6 could avoid 26% of unnecessary prostate biopsy, the overall biopsy rate was 20%, and the sensitivity and negative predictive value were 93% and 93%, respectively. 89%, and the missed diagnosis rate of ≥GG2 grade prostate cancer was 7%. Subsequently, in 2020, Mckiernan et al. (55) further evaluated the diagnostic power of the ExoDx Prostate Intelliscore in patients with repeat biopsy in a cohort study (n=229), and the EPI score in patients with aggressive PCa whose diagnosis predicted a negative initial biopsy Better than ERSPC and serum PSA risk prediction model, when the critical value of EPI score is 15.6, the negative predictive value is 92%, the AUC is 0.66 (95% CI: 0.55-0.78), 26% of unnecessary biopsies can be avoided, and only missed diagnosis 2.6% aggressive PCa. In this study, 71.6% of patients were Caucasian and 14.4% were African-American, which is the most diverse study of ethnic groups, but there are no studies on Asian populations.

Notably, Gleason score (GS) may be a potential source of heterogeneity. Gleason score is one of the important indicators for judging the prognosis of prostate cancer (56). The increase of GS score is significantly associated with recurrence, distant metastasis and PCa death, while the decrease of GS score is a protective factor (57). Some of the studies included in this study focused on this, for example, Tomlins SA et al. (58) of the University of Michigan developed a novel PCa diagnostic prediction model (MiPS), which was predictive of aggressive PCa (Gleason score ≥ 7) AUC was 0.77, much better than serum PSA alone (AUC: 0.651). There is still a lot of research on urine markers that needs to focus on this in the future.

### 4.1 Limitations

This study systematically compared the accurate performance of four commonly used urine markers and serum PSA screening tools in prostate cancer patients, but there are some limitations. ①There are few literatures included in some urine markers. For example, there is only one literature about ExoDx Prostate Intelliscore, which has a certain degree of bias; ②The study found that some results have obvious heterogeneity, which may be due to different urine collection methods. And sample processing; ③Some studies did not report the required outcome indicators in detail, but were estimated based on known variables, which may have had a certain impact on the ranking of the final diagnostic performance. ④The inconsistency of the cutoff values of urine markers such as PCA3 and PSA in the included literature may have a selection bias to a certain extent.

## 5 Conclusions

Overall, this study compared the diagnostic performance of four urine markers and serum PSA screening tools in prostate cancer screening based on Bayesian network meta-analysis. The results showed that the sensitivity of Selectmdx, MIPS, Specificity, positive predictive value, negative predictive value and accuracy are good, and it can be used as the most suitable urine marker for prostate cancer screening and diagnosis. Although PCa urine markers have certain limitations, they represent a valuable non-invasive cancer detection method, which has important clinical significance in identifying invasive PCa at an early stage, reducing unnecessary needle biopsies, and reducing the waste of medical resources. In the future, more rigorously designed, large-scale, multi-center studies are still needed to further explore the application value of different urinary markers in the screening of PCa patients.

## Data availability statement

The original contributions presented in the study are included in the article/**Supplementary Material**. Further inquiries can be directed to the corresponding authors.

## Author contributions

All authors: Study concept and design, critical revision of the manuscript, and interpretation of data. LW, WH, GS, GZ, ZC, FX, WT, and XZ: Data acquisition and management and statistical analysis. LW and CM: drafting of the manuscript. GS: study supervision. All authors contributed to the article and approved the submitted version.

## Funding

Science and Technology Foundation of Guizhou Provincial Health Commission (Reference number: Gzwkj2023-373).

## Acknowledgments

We thank all the reviewers for their assistance and support.

## Conflict of interest

The authors declare that the research was conducted in the absence of any commercial or financial relationships that could be construed as a potential conflict of interest.

## Publisher’s note

All claims expressed in this article are solely those of the authors and do not necessarily represent those of their affiliated organizations, or those of the publisher, the editors and the reviewers. Any product that may be evaluated in this article, or claim that may be made by its manufacturer, is not guaranteed or endorsed by the publisher.

## References

[1] Siegel RL Miller KD Fuchs HE Jemal A . Cancer statistics, 2021. CA: Cancer J Clin (2021) 71(1):7–33. doi: 10.3322/caac.21654 33433946

[2] Bahmad HF Jalloul M Azar J Moubarak MM Samad TA Mukherji D . Tumor microenvironment in prostate cancer: Toward identification of novel molecular biomarkers for diagnosis, prognosis, and therapy development. Front Genet (2021) 12:652747. doi: 10.3389/fgene.2021.652747 33841508 PMC8033163

[3] Snider AJ Seeds MC Johnstone L Snider J M Hallmark B Dutta R . Identification of plasma glycosphingolipids as potential biomarkers for prostate cancer (PCa) status. Biomolecules (2020) 10(10):1393. doi: 10.3390/biom10101393 33007922 PMC7600119

[4] Merriel SWD Funston G Hamilton W . Prostate cancer in primary care. Adv Ther (2018) 35(9):1285–94. doi: 10.1007/s12325-018-0766-1 PMC6133140 30097885

[5] Brawley S Mohan R Nein CD . Localized prostate cancer: Treatment options. Am Family physician (2018) 97(12):798–805. Available at: https://www.aafp.org/pubs/afp/issues/2018/0615/p798.html. 30216009

[6] Gan J Zeng X Wang X Wu Y Lei P Wang Z . Effective diagnosis of prostate cancer based on mRNAs from urinary exosomes. Front Med (Lausanne) (2022) 9:736110. doi: 10.3389/fmed.2022.736110 35402423 PMC8983915

[7] Carlsson SV Vickers AJ . Screening for prostate cancer. Med Clinics North America (2020) 104(6):1051–62. doi: 10.1016/j.mcna.2020.08.007 PMC8287565 33099450

[8] Tan GH Nason G Ajib K Woon DTS Herrera-Caceres J Alhunaidi O . Smarter screening for prostate cancer. World J Urol (2019) 37(6):991–9. doi: 10.1007/s00345-019-02719-5 30859272

[9] Mugoni V Ciani Y Nardella C Demichelis F . Circulating RNAs in prostate cancer patients. Cancer Lett (2022) 524:57–69. doi: 10.1016/j.canlet.2021.10.011 34656688

[10] Kim WT Yun SJ Kim WJ . For physicians managing voiding dysfunction, improving the detection rate of early prostate cancer and discrimination from benign prostatic hyperplasia, in a molecular biomarker aspects. Int neurourology J (2019) 23(1):5–12. doi: 10.5213/inj.1836262.131 PMC6449661 30943689

[11] Eskra JN Rabizadeh D Pavlovich CP Catalona WJ Luo J . Approaches to urinary detection of prostate cancer. Prostate Cancer prostatic Dis (2019) 22(3):362–81. doi: 10.1038/s41391-019-0127-4 PMC6640078 30655600

[12] Kim JH Hong SK . Clinical utility of current biomarkers for prostate cancer detection. Investig Clin Urol (2021) 62(1):1–13. doi: 10.4111/icu.20200395 PMC7801171 33381926

[13] Mistry K Cable G . Meta-analysis of prostate-specific antigen and digital rectal examination as screening tests for prostate carcinoma. J Am Board Family Pract (2003) 16(2):95–101. doi: 10.3122/jabfm.16.2.95 12665174

[14] Hessels D Schalken JA . Urinary biomarkers for prostate cancer: a review. Asian J andrology (2013) 15(3):333–9. doi: 10.1038/aja.2013.6 PMC3739649 23524531

[15] Liu J Li Y Yang D Yang C Mao L . Current state of biomarkers for the diagnosis and assessment of treatment efficacy of prostate cancer. Discovery Med (2019) 27(150):235–43. Available at: https://www.discoverymedicine.com/Jihai-Liu/2019/06/biomarkers-for-diagnosis-and-assessment-of-treatment-efficacy-of-prostate-cancer/. 31421692

[16] Ballesteros Ruiz C Álvarez-Maestro M Aguilera Bazán A Martínez-Piñero L . [Urinary biomarkers in the diagnosis of prostate cancer.]. Archivos espanoles urologia (2022) 75(2):165–72. Avalable at: https://www.aeurologia.com/EN/Y2022/V75/I2/165. 35332886

[17] Yang A Pechlivanoglou P Aoyama K . Interpreting and assessing confidence in network meta-analysis results: an introduction for clinicians. J Anesth (2022) 36(4):524–31. doi: 10.1007/s00540-022-03072-5 PMC9338903 35641661

[18] Yang B Mallett S Takwoingi Y Davenport CF Hyde CJ Whiting PF . QUADAS-c: A tool for assessing risk of bias in comparative diagnostic accuracy studies. Ann Internal Med (2021) 174(11):1592–9. doi: 10.7326/M21-2234 34698503

[19] Salanti G Ades AE Ioannidis JP . Graphical methods and numerical summaries for presenting results from multiple-treatment meta-analysis: an overview and tutorial. J Clin Epidemiol (2011) 64(2):163–71. doi: 10.1016/j.jclinepi.2010.03.016 20688472

[20] Chaimani A Higgins JP Mavridis D Spyridonos P Salanti G . Graphical tools for network meta-analysis in STATA. PloS One (2013) 8(10):e76654. doi: 10.1371/journal.pone.0076654 24098547 PMC3789683

[21] Marotta N Demeco A Moggio L Marinaro C Pino I Barletta M . Comparative effectiveness of breathing exercises in patients with chronic obstructive pulmonary disease. Complementary therapies Clin Pract (2020) 41:101260. doi: 10.1016/j.ctcp.2020.101260 33221632

[22] Hao Z Zhang X Chen P . Effects of ten different exercise interventions on motor function in parkinson's disease patients-a network meta-analysis of randomized controlled trials. Brain Sci (2022) 12(6). doi: 10.3390/brainsci12060698 PMC9221238 35741584

[23] Goode RR Marshall SJ Duff M Chevli E Chevli KK . Use of PCA3 in detecting prostate cancer in initial and repeat prostate biopsy patients. Prostate (2013) 73(1):48–53. doi: 10.1002/pros.22538 22585386

[24] Tinzl M Marberger M Horvath S Chypre C . DD3PCA3 RNA analysis in urine–a new perspective for detecting prostate cancer. Eur Urol (2004) 46(2):182–6. doi: 10.1016/j.eururo.2004.06.004 15245811

[25] Roobol MJ Schröder FH Van Leeuwen P Wolters T Van Den Bergh RC Van Leenders GJ . Performance of the prostate cancer antigen 3 (PCA3) gene and prostate-specific antigen in prescreened men: exploring the value of PCA3 for a first-line diagnostic test. Eur Urol (2010) 58(4):475–81. doi: 10.1016/j.eururo.2010.06.039 20637539

[26] De La Taille A Irani J Graefen M Chun F De Reijke T Kil P . Clinical evaluation of the PCA3 assay in guiding initial biopsy decisions. J Urol (2011) 185(6):2119–25. doi: 10.1016/j.juro.2011.01.075 21496856

[27] Vlaeminck-Guillem V Campos-Fernandes JL Champetier D Chikh K Decaussin-Perucci M Devonec M . [Value of PCA3 urinary test for prostate biopsy decision: the Lyon-sud university hospital experience]. Annales biologie clinique (2011) 69(1):31–9. doi: 10.1684/abc.2010.0513 21463993

[28] Ng CF Yeung R Chiu PK Lam NY Chow J Chan B . The role of urine prostate cancer antigen 3 mRNA levels in the diagnosis of prostate cancer among Hong Kong Chinese patients. Hong Kong Med J (2012) 18(6):459–65. Avalable at: https://www.hkmj.org/abstracts/v18n6/459.htm. 23223645

[29] Ouyang B Bracken B Burke B Chung E Liang J Ho SM . A duplex quantitative polymerase chain reaction assay based on quantification of alpha-methylacyl-CoA racemase transcripts and prostate cancer antigen 3 in urine sediments improved diagnostic accuracy for prostate cancer. J Urol (2009) 181(6):2508–13. doi: 10.1016/j.juro.2009.01.110 PMC4372725 19371911

[30] Ramos CG Valdevenito R Vergara I Anabalon P Sanchez C Fulla J . PCA3 sensitivity and specificity for prostate cancer detection in patients with abnormal PSA and/or suspicious digital rectal examination. First Latin American experience. Urologic Oncol (2013) 31(8):1522–6. doi: 10.1016/j.urolonc.2012.05.002 22687565

[31] Van Gils MP Hessels D Van Hooij O Jannink SA Peelen WP Hanssen SL . The time-resolved fluorescence-based PCA3 test on urinary sediments after digital rectal examination; A Dutch multicenter validation of the diagnostic performance. Clin Cancer Res (2007) 13(3):939–43. doi: 10.1158/1078-0432.CCR-06-2679 17289888

[32] Fradet Y Saad F Aprikian A Dessureault J Elhilali M Trudel C . uPM3, a new molecular urine test for the detection of prostate cancer. Urology (2004) 64(2):311–5. doi: 10.1016/j.urology.2004.03.052 15302485

[33] Mckiernan J Donovan MJ Margolis E Partin A Carter B Brown G . A prospective adaptive utility trial to validate performance of a novel urine exosome gene expression assay to predict high-grade prostate cancer in patients with prostate-specific antigen 2-10ng/ml at initial biopsy. Eur Urol (2018) 74(6):731–8. doi: 10.1016/j.eururo.2018.08.019 30237023

[34] Sanda MG Feng Z Howard DH Tomlins SA Sokoll LJ Chan DW . Association between combined TMPRSS2:ERG and PCA3 RNA urinary testing and detection of aggressive prostate cancer. JAMA Oncol (2017) 3(8):1085–93. doi: 10.1001/jamaoncol.2017.0177 PMC5710334 28520829

[35] Salami SS Schmidt F Laxman B Regan MM Rickman DS Scherr D . Combining urinary detection of TMPRSS2:ERG and PCA3 with serum PSA to predict diagnosis of prostate cancer. Urologic Oncol (2013) 31(5):566–71. doi: 10.1016/j.urolonc.2011.04.001 PMC3210917 21600800

[36] Van Neste L Hendriks RJ Dijkstra S Trooskens G Cornel EB Jannink SA . Detection of high-grade prostate cancer using a urinary molecular biomarker-based risk score. Eur Urol (2016) 70(5):740–8. doi: 10.1016/j.eururo.2016.04.012 27108162

[37] Busetto GM Del Giudice F Maggi M De Marco F Porreca A Sperduti I . Prospective assessment of two-gene urinary test with multiparametric magnetic resonance imaging of the prostate for men undergoing primary prostate biopsy. World J Urol (2021) 39(6):1869–77. doi: 10.1007/s00345-020-03359-w PMC8217060 32681273

[38] Fiorella D Marenco JL Mascarós JM Borque-Fernando Á Esteban LM Calatrava A . Role of PCA3 and SelectMDx in the optimization of active surveillance in prostate cancer. Actas urologicas espanolas (2021) 45(6):439–46. doi: 10.1016/j.acuroe.2020.10.013 34148844

[39] Urabe F Ochiya T Egawa S . Re: A prospective adaptive utility trial to validate performance of a novel urine exosome gene expression assay to predict high-grade prostate cancer in patients with prostate-specific antigen 2-10ng/ml at initial biopsy. Eur Urol (2019) 76(2):254–5. doi: 10.1016/j.eururo.2019.02.036 30905514

[40] Leyten GH Hessels D Smit FP Jannink SA De Jong H Melchers WJ . Identification of a candidate gene panel for the early diagnosis of prostate cancer. Clin Cancer Res (2015) 21(13):3061–70. doi: 10.1158/1078-0432.CCR-14-3334 25788493

[41] Maggi M Del Giudice F Falagario UG Cocci A Russo GI Di Mauro M . SelectMDx and multiparametric magnetic resonance imaging of the prostate for men undergoing primary prostate biopsy: A prospective assessment in a multi-institutional study. Cancers (2021) 13(9). doi: 10.3390/cancers13092047 PMC8122883 33922626

[42] Dijkstra S Govers TM Hendriks RJ Schalken JA Van Criekinge W Neste L . Cost-effectiveness of a new urinary biomarker-based risk score compared to standard of care in prostate cancer diagnostics - a decision analytical model. BJU Int (2017) 120(5):659–65. doi: 10.1111/bju.13861 28370948

[43] Tomlins SA Rhodes DR Perner S Dhanasekaran SM Mehra R Sun XW . Recurrent fusion of TMPRSS2 and ETS transcription factor genes in prostate cancer. Sci (New York NY) (2005) 310(5748):644–8. doi: 10.1126/science.1117679 16254181

[44] Magi-Galluzzi C Tsusuki T Elson P Simmerman K Lafargue C Esgueva R . TMPRSS2-ERG gene fusion prevalence and class are significantly different in prostate cancer of Caucasian, African-American and Japanese patients. Prostate (2011) 71(5):489–97. doi: 10.1002/pros.21265 20878952

[45] Hessels D Klein Gunnewiek JM Van Oort I Karthaus HF Van Leenders GJ Van Balken B . DD3(PCA3)-based molecular urine analysis for the diagnosis of prostate cancer. Eur Urol (2003) 44(1):8–15. doi: 10.1016/S0302-2838(03)00201-X 12814669

[46] Wei JT . Urinary biomarkers for prostate cancer. Curr Opin Urol (2015) 25(1):77–82. doi: 10.1097/MOU.0000000000000133 25405933 PMC4270695

[47] Robert G Jannink S Smit F Aalders T Hessels D Cremers R . Rational basis for the combination of PCA3 and TMPRSS2:ERG gene fusion for prostate cancer diagnosis. Prostate (2013) 73(2):113–20. doi: 10.1002/pros.22546 22674214

[48] Matuszczak M Schalken JA Salagierski M . Prostate cancer liquid biopsy biomarkers' clinical utility in diagnosis and prognosis. Cancers (2021) 13(13):3373. doi: 10.3390/cancers13133373 34282798 PMC8268859

[49] Marks LS Fradet Y Deras IL Blase A Mathis J Aubin SM . PCA3 molecular urine assay for prostate cancer in men undergoing repeat biopsy. Urology (2007) 69(3):532–5. doi: 10.1016/j.urology.2006.12.014 17382159

[50] Lee D Shim SR Ahn ST Oh MM Moon DG Park HS . Diagnostic performance of the prostate cancer antigen 3 test in prostate cancer: Systematic review and meta-analysis. Clin genitourinary Cancer (2020) 18(5):402–8.e5. doi: 10.1016/j.clgc.2020.03.005 32280028

[51] Rodríguez SVM García-Perdomo HA . Diagnostic accuracy of prostate cancer antigen 3 (PCA3) prior to first prostate biopsy: A systematic review and meta-analysis. Can Urological Assoc J (2020) 14(5):E214–e9. doi: 10.5489/cuaj.6008 PMC7197956 31793864

[52] Salciccia S Capriotti AL Laganà A Fais S Logozzi M De Berardinis E . Biomarkers in prostate cancer diagnosis: From current knowledge to the role of metabolomics and exosomes. Int J Mol Sci (2021) 22(9). doi: 10.3390/ijms22094367 PMC8122596 33922033

[53] Mckiernan J Donovan MJ O’Neill V Bentink S Noerholm M Belzer S . A novel urine exosome gene expression assay to predict high-grade prostate cancer at initial biopsy. JAMA Oncol (2016) 2(7):882–9. doi: 10.1001/jamaoncol.2016.0097 27032035

[54] Donovan MJ Noerholm M Bentink S Belzer S Skog J O’Neill V . A molecular signature of PCA3 and ERG exosomal RNA from non-DRE urine is predictive of initial prostate biopsy result. Prostate Cancer prostatic Dis (2015) 18(4):370–5. doi: 10.1038/pcan.2015.40 26345389

[55] Mckiernan J Noerholm M Tadigotla V Kumar S Torkler P Sant G . A urine-based exosomal gene expression test stratifies risk of high-grade prostate cancer in men with prior negative prostate biopsy undergoing repeat biopsy. BMC Urol (2020) 20(1):138. doi: 10.1186/s12894-020-00712-4 32873277 PMC7466797

[56] Sehn JK . Prostate cancer pathology: Recent updates and controversies. Missouri Med (2018) 115(2):151–5. PMC6139855 30228708

[57] Wang X Zhang Y Ji Z Yang P Tian Y . Old men with prostate cancer have higher risk of Gleason score upgrading and pathological upstaging after initial diagnosis: a systematic review and meta-analysis. World J Surg Oncol (2021) 19(1):18. doi: 10.1186/s12957-021-02127-3 33472645 PMC7818761

[58] Tomlins SA Day JR Lonigro RJ Hovelson DH Siddiqui J Kunju LP . Urine TMPRSS2:ERG plus PCA3 for individualized prostate cancer risk assessment. Eur Urol (2016) 70(1):45–53. doi: 10.1016/j.eururo.2015.04.039 25985884 PMC4644724

