# Supplementary material for: Accuracy of novel urinary biomarker tests in the diagnosis of prostate cancer: A systematic review and network meta-analysis
Source: Front Oncol. 2022 Nov 15;12:1048876. doi: 10.3389/fonc.2022.1048876 (PMC9706202; doi:10.3389/fonc.2022.1048876)
Supplement: Supplementary file 1 [file Table_1.docx]

| #1 | "ProstaticNeoplasms"[Mesh] |
| --- | --- |
| #2 | (((((((((((((((((ProstateNeoplasms[Title/Abstract])OR(Neoplasms,Prostate[Title/Abstract]))OR(Neoplasm,Prostate[Title/Abstract]))OR(ProstateNeoplasm[Title/Abstract]))OR(Neoplasms,Prostatic[Title/Abstract]))OR(Neoplasm,Prostatic[Title/Abstract]))OR(ProstaticNeoplasm[Title/Abstract]))OR(ProstateCancer[Title/Abstract]))OR(Cancer,Prostate[Title/Abstract]))OR(Cancers,Prostate[Title/Abstract]))OR(ProstateCancers[Title/Abstract]))OR(CanceroftheProstate[Title/Abstract]))OR(ProstaticCancer[Title/Abstract]))OR(Cancer,Prostatic[Title/Abstract]))OR(Cancers,Prostatic[Title/Abstract]))OR(ProstaticCancers[Title/Abstract]))OR(CancerofProstate[Title/Abstract]))) |
| #3 | #1OR#2 |
| #4 | Urinarybiomarkers |
| #5 | (ProgensaPCA3)OR(ExoDxProstateIntelliscore)OR(SelectMDx)OR(Mi-ProstateScore) |
| #6 | #4AND#5 |
| #7 | #3AND#6 |

Supplementary Table1. Search strategy on PubMed.
